# Relationship Between Depression and Subtypes of Early Life Stress in Adult Psychiatric Patients

**DOI:** 10.3389/fpsyt.2019.00019

**Published:** 2019-02-05

**Authors:** Camila Maria Severi Martins-Monteverde, Cristiane Von Werne Baes, Emilene Reisdorfer, Thalita Padovan, Sandra Marcia de Carvalho Tofoli, Mario Francisco Juruena

**Affiliations:** ^1^Stress and Affective Disorders Programme, University of São Paulo, São Paulo, Brazil; ^2^School of Community and Health Studies, Centennial College, Toronto, ON, Canada; ^3^Centre for Affective Disorders, Department Psychological Medicine, King's College London, London, United Kingdom

**Keywords:** early life stress, subtypes of early life stress, depressive disorder, emotional abuse, neglect, childhood trauma

## Abstract

Numerous studies have researched the aggravating and maintainer effect of Early Life Stress in patients adults with psychiatric disorders. This study examined the relationship between depression and subtypes of early life stress among 81 psychiatric patients treated at the inpatient Day Hospital Unit of a University General Hospital. Psychiatric diagnosis was confirmed according to the MINI International Neuropsychiatric Interview (MINI). The Childhood Trauma Questionnaire (CTQ) was used for evaluating as retrospective assessment of the presence of ELS on these patients, and we also evaluated the severity of hopelessness with the Beck Hopelessness Scale (BHS). Our results suggested that the occurrence of depression in adulthood is related to situations of emotional abuse, sexual, and physical neglect during childhood. The analysis between depression and childhood emotional abuse was significant after a multiple logistic regression analysis OR (IC 95%): 4.4 (1.7–11.2), even accounting for gender adjusted OR [AOR] 4.0; (IC 1.5–10.5); psychiatry family history AOR 3.8 (1.4–10.5); previous suicide attempted AOR 3.7; (1.4–10.5) and Hopelessness AOR 3.2 (1.11–9.4). Thus, these findings demonstrate emotional abuse as a significant risk factor to be part of the mechanism involved in the pathogenesis of depression related to early life stress.

## Introduction

Recent studies have examined the effect of stress in the early stages of development of the individual demonstrating that when stress occurs early, can lead to “biological scars” lifelong (1). The Early Life Stress (ELS) is a variety of traumatic experiences that can occur during childhood and adolescence, such as abuse, neglect, parental loss, divorce of parents, caregivers with psychiatric disorders, childhood disease involving prolonged hospitalizations, lack of primary care, abandonment, deprivation of food, and adequate shelter, lack of encouragement and support, as well as family violence (2, 3). Numerous studies have studied the aggravating effect and maintainer of the ELS in psychiatric disorders in adults. Recognized studies (4–7) evidence the future impact of various early trauma on mental health. In the systematic review we conducted (8) the data demonstrated that individual exposure to adversities in childhood and adolescence is predictive of psychiatric disorders in adulthood, such as depression and borderline personality disorder. In this same direction, other systematic review evaluated the association between subtypes of ELS with psychiatric disorders in adults, finding that physical abuse, sexual, and unspecified neglect are associated with mood disorder and anxiety disorders, while emotional abuse is associated with personality disorder and schizophrenia and that the physical neglect is related to personality disorders (9). In consonance, some studies (10–12) showed that emotional abuse is associated with depressive symptoms in adults. Furthermore, the literature indicates that it is quite common co-occurrence of abuse or neglect, it is difficult occurs in a single subtype of ELS, so that the incidence may range around 40–95% of the sample (13, 14). The study by Chartier et al. (15), shown that among psychiatric patients with ELS, 37% of them had reports of the occurrence of more than one subtype of ELS. The study of Felitti et al. (16) showed that individuals who have experienced situations of child adversity likely to have experienced more than one subtype ELS varies around approximately 80%. According to Hahm et al. (17), the co-occurrence of ELS subtypes is an essential measure of the severity of psychopathology, since in many studies was associated with increased severity of psychiatric symptoms. In this sense, given the importance of stress in the development of psychiatric disorders, recently the Diagnostic and Statistical Manual of Mental Disorders (DSM-5), included among its ratings a specific category for the diagnosis of Trauma and Stressor-Related Disorders (18). Thus, researchers also point to the need to analyze how stress triggers interact with psychopathology, showing that stress increases the risk for depression, but leave the individual susceptible to the occurrence of different types of stress (10). According to Heim and Nemeroff (19) children exposed to traumatic experiences in early childhood have an increased risk of developing depression. The persistent sensitization of the circuits of the central nervous system, which are integrally involved in the regulation of tension and emotion, may represent the underlying biological substrates to increased vulnerability to subsequent stress, predisposing these individuals to develop a broad range of physical and mental disorders which are known to express or worsen in relation to acute stress. The intense stress starts to play a substantial adverse effect on a person's life, affecting not only the mental health but physical, cognition, and occupational performance in everyday activities. Another important consequence of ELS is related to the increase of suicide attempts in individuals with psychiatric disorders who suffered ELS. Several studies have suggested an association between ELS and increased suicidality in adulthood (7, 20–24). The study by Sfoggia et al. (25) it was shown that adults with a history of severe abuse and neglect had more suicide attempts than those who reported no ELS. Also, in the survey conducted by Brown et al. (26) relevant data found, in which victims of sexual abuse has eight times more likely to suicide. Our previous data (27), demonstrated that among the ELS subtypes, significant association was found only between the emotional abuse subtype and psychiatric diagnoses. Emotional abuse was positively associated with psychopathology in adults, particularly with depressive disorders. The patients with a history of emotional abuse had higher severity in depression, hopelessness, suicidal ideation, anxiety, and impulsivity. Thus, the present study aimed to analyze the relationship between depression and subtypes of ELS among patients with psychiatric disorders, mainly control the analysis of the association between emotional abuse and patients with depression treated at the inpatient Day Hospital Unit of a General University Hospital.

## Methods

### Study Sample and Design

The sample was composed of 81 adult psychiatric patients, treated at inpatient Day Hospital Unit of a General University Hospital. The inclusion criteria for this study were patients with psychiatric diagnosis confirmed according to Mini International Neuropsychiatric Interview (MINI) (28, 29), in the follow-up inpatient Day Hospital Unit, aged 18–65 years. We excluded patients with any significant physical illness in acute, mental disorders caused by a general medical condition or resulting from the direct physiological effect of a substance, substance-related disorders, mental retardation, and patients in an acute psychotic episode, cognitive deficits, and neurological progressive and degenerative diseases.

### Procedure and Measures

The study was approved by the Research Ethics Committee of the General University Hospital, Faculty of Medicine of Ribeirao Preto, University of Sao Paulo. Participants were informed that the purpose of the study was to investigate the association between early life stress and psychiatric disorders in adult life. Later, written informed consent was obtained from all patients, and the questionnaires included in this study were applied.

### Demographic and Clinical Data

Clinical and sociodemographic characteristics (age, gender, religious practice, family history of psychiatric disorder, suicide attempt, among others) were obtained through the administration of a sociodemographic questionnaire developed by researchers. We also evaluated the severity of hopelessness with the Beck Hopelessness Scale [BHS; (30)]. This is a self-report questionnaire with 20 true-false statements developed to assess the extent of positive and negative beliefs about the future. The version in Portuguese was translated and adapted by Cunha (31).

### Psychiatric Diagnosis

The assessment of psychiatric diagnosis was conducted using Mini International Neuropsychiatric Interview [MINI; (28)], the version in Portuguese translated and adapted by Amorim (29). The MINI is a brief structured interview designed to assess criteria for the majority of psychiatric disorders classified in DSM-IV and ICD-10. There is a version called MINI PLUS, intended for assessment of the primary psychiatric diagnosis throughout life, in clinical and research psychiatry and systematically explores all the criteria for inclusion and exclusion and chronology (onset and duration of the disorder, number of episodes) (29). All subjects were interviewed by two senior psychiatrists (MFJ; CWB) trained and certified to the use of the standardized interviews. The interviewers had long-standing experience in the administration of standardized interviews. In the total sample, the distribution of psychiatric disorders was as follows: more than 75% had a diagnosis of mood disorders, prevailing depressive disorders (*n* = 44; 54%), followed by bipolar disorder (*n* = 17; 21%). The other diagnoses assessed were anxiety disorders (*n* = 10; 12.3%), eating disorders (*n* = 3; 4%), and others (*n* = 7; 8%). We did not include patients with diagnoses of substance use disorder in the sample because the Day Hospital Unit does not admit patients with this diagnosis.

### Subtypes of Early Life Stress

The subtypes of ELS was assessed using the Childhood Trauma Questionnaire [CTQ; (32)]. The CTQ is a retrospective self-report questionnaire that investigates the history of abuse and neglect during childhood and can be applied to adolescents (from 12 years) and adults, where the responder assigns values of frequency to 28 sentences, graduate issues related to situations arising in childhood. The CTQ evaluates five subtypes of early life stress:

- Emotional abuse: verbal assaults on a child's sense of worth or well-being or any humiliating or demeaning behavior directed toward a child by an adult or older person;
- Physical abuse: bodily assaults on a child by an adult or older person that posed a risk of or resulted in injury;
- Sexual abuse: sexual contact or conduct between a child younger than 18 years of age and an adult or older person;
- Emotional neglect: the failure of caretakers to meet children's basic emotional and psychological needs, including love, belonging, nurturance, and support;
- Physical neglect: the failure of caretakers to provide for a child's basic physical needs, including food, shelter, clothing, safety, and healthcare (inadequate parental supervision was also included in this definition if it placed children's security in jeopardy) (3).

The items are rated on a Likert scale ranging from 1 (never) to 5 (very often). Furthermore, the scores range from 5 to 25 for each subtype of ELS. A cut-point for early life stress, and its subtypes (emotional, physical and sexual abuse, and emotional and physical neglect), was defined as when one of these experiences before the age of 18 reached a degree of at least moderate to severe, according to classification in the CTQ (3, 32–34). The version in Portuguese was translated and adapted by Grassi-Oliveira et al. (35).

### Statistical Analysis

Logistic multiple regression analysis was conducted in Stata 9 statistical program to test the relationship between depression and emotional abuse when controlled by independent variables. Univariate analysis of depression was performed with each of the analyzed independent variables. We conducted the analysis of association between ELS and all psychiatric disorders. However, according to data presented in Table 2, there was no significant difference between the groups with and with- out ELS (χ^2^ = 8.44, df = 6.0, *p* = 0.188) in relation to the distribution of psychiatric diagnoses.

In addition, we performed a second statistical analysis, comparing ELS subtypes with psychiatric disorders. Thus, significant association was found only between the emotional abuse subtype and psychiatric diagnoses. Those with *p* < 0.30 or that are relevant in the literature to the Raw Model: Depression Disorder + Emotional Abuse and Model 2 = Raw Model + Gender; Model 3 = Model 2 + Familiar History of Mental Disorders; Model 4 = Model 3 + Suicide Attempt; and Model 5 = Model 4 + Hopelessness; were included in the multiple models using the stepwise forward strategy when the association between depression and emotional abuse in childhood was adjusted for all independent variables. Remained for analysis the variables with *p* < 0.20 or those that adjusted the dependent variables. We also used Chi-square for analysis of the occurrence of subtypes of ELS and the Student *t*-test for analysis of CTQ scores.

## Results

The participants were predominantly women (72.8%), under 40 years of age, reporting religious practice (86.5%) and with a family history of mental disorder (77.0%). Concerning, the psychiatric diagnosis, 54% of patients seen at the hospital had depression, followed by Bipolar disorder (21.0%). The other diagnoses assessed were: Anxiety Disorders (12.3%), Schizophrenia and Other Psychotic Disorders (6.2%), Eating Disorders (3.7%), Dissociative Disorders (1.2%), Impulse Control Disorders not elsewhere classified (1.2%). We do not include patients with diagnoses of Substance Use Disorder in the sample because the University Day Hospital does not include patients with this diagnosis. Further details can be found in Table 1.

**Table 1:** Demographic and clinical characteristics of the sample.

**Variable**	***N* (%)**
**GENDER**	
Male	22 (27.2)
Female	59 (72.8)
**AGE GROUP**	
< 29 yrs	22 (27.2)
30–39 yrs	22 (27.2)
40–49 yrs	29 (35.8)
>50 yrs	8 (9.8)
**RELIGIOUS PRACTICE**	
Yes	64 (86.5)
No	10 (13.5)
**MARITAL STATUS**	
With partner	41 (50.6)
Without partner	40 (49.4)
**SUICIDE ATTEMPT**	
Yes	54 (66.7)
No	27(33.3)
**FAMILY HISTORY OF PSYCHIATRIC DISORDER**	
Yes	57 (77.0)
No	17 (23.0)
**DEPRESSION DISORDER**	
Yes	44 (54.3)
No	37 (45.7)

The data regarding the subtypes of early life stress are shown in Table 2. There has been a higher frequency of childhood emotional abuse among patients, while the sexual abuse had a lower occurrence, see in Table 3.

**Table 2:** Occurrence and CTQ scores of subtypes of early life stress among the whole sample (*n* = 81).

**Variable**	***N* (%)**	***p* (*X*^**2**^)**	**CTQ score, mean ± SEM**	***p* (*t*)**
Childhood emotional abuse	–	< 0.001	–	< 0.001
No	35 (43.2)	–	8.26 (±0.40)	–
Yes	46 (56.8)	–	16.7 (±0.68)	–
Childhood physical abuse	–	< 0.001	–	< 0.001
No	49 (60.5)	–	5.60 (±0.22)	–
Yes	32 (39.5)	–	11.96 (±0.80)	–
Childhood sexual abuse	–	< 0.001	–	< 0.001
No	59 (72.8)	–	5.04 (±0.04)	–
Yes	22 (27.2)	–	9.25 (±0.84)	–
Childhood emotional neglect	–	< 0.001	–	< 0.001
No	44 (54.3)	–	9.43 (±0.59)	–
Yes	37 (45.7)	–	16.32 (±0.68)	–
Childhood physical neglect	–	< 0.001	–	< 0.001
No	42 (51.9)	–	6.43 (±0.31)	–
Yes	39 (48.2)	–	11.79 (±0.53)	–

*CTQ subscale, threshold score of each subtype is Emotional Abuse score > 13; Physical Abuse score > 10; Sexual Abuse score > 8; Emotional Neglect > 15; Physical Neglect >10*.

**Table 3:** Relationship between depression and subtypes of early life stress among patients.

**Variable**	**Depression**		***p*-value**
	**No 37 (45.7%)**	**Yes 44 (54.3%)**	
Childhood emotional abuse			**0.002**
No	53 (65.7)	28 (34.3)	
Yes	25 (30.4)	56 (69.6)	
Childhood physical abuse			0.23
No	41(51.1)	40 (48.9)	
Yes	30 (37.5)	51 (62.5)	
Childhood sexual abuse			**0.01**
No	44 (54.2)	37 (45.8)	
Yes	18 (22.7)	63 (77.3)	
Childhood emotional neglect			0.19
No	42 (52.3)	39 (47.7)	
Yes	31 (37.8)	50 (62.2)	
Childhood physical neglect			0.08
No	44 (54.8)	37 (45.2)	
Yes	29 (35.9)	52 (64.1)	

In this population, depression was associated with emotional abuse and sexual abuse, with considerable statistical significance. However, the relationship with physical neglect, demonstrated a trend (*p* = 0.08). These data indicate that the occurrence of depression in adulthood is related to situations of emotional abuse, sexual and physical neglect during childhood.

The analysis of the chances of occurrence of depression according to the epidemiological characteristics showed that women are more exposed compared with men. Moreover, it was also observed an increase of about four times the chance of depression among people who have suffered emotional or sexual abuse in childhood or who reported at least one suicide attempt throughout life. Patients with a high level of hopelessness also showed a higher chance of depression (Table 4).

**Table 4:** Univariate logistic regression between depression and childhood emotional abuse among patients.

**Variable**	**Odds ratio (IC 95%)**	***p*-value**
Gender		0.14
Male	1	
Female	2.11 (0.78–5.71)	
Age group		
< 29 yrs	1	
30–39 yrs	1.73 (0.53–5.82)	0.36
40–49 yrs	2.74 (0.87–8.61)	0.08
>50 yrs	1.44 (0.28–7.34)	0.65
Religious practice		0.31
Yes	1	
No	1.24 (0.32–4.83)	
Marital status		0.90
With partner	1	
Without partner	1.05 (0.44–2.53)	
Family history of mental disorder		0.45
No	1	
Yes	0.65 (0.21–1.99)	
Childhood emotional abuse		**0.002**
No	1	
Yes	4.38 (1.71–11.2)	
Childhood sexual abuse		**0.01**
No	1	
Yes	4.03 (1.31–12.36)	
Suicide attempt		**0.009**
No	1	
Yes	3.68 (1.39–9.77)	
Hopelessness (bhs)		**0.01**
Normal or Suave Hopelessness	1	
Moderate/Severe Hopelessness	3.07 (1.20–7.87)	

Patients with a history of childhood emotional abuse had a frequency of 4.38 higher of depression compared to those without such a history in the bivariate regression (Table 5). In the following models, with adjustments for demographic variables and health conditions, the magnitude of odds ratio decreased, although the adjusted odds ratio was still 3.23 times higher in the group exposed to emotional abuse during childhood (*p*-value 0.031). Multiple Logistic regression analysis between depression and childhood emotional abuse was significant after a multiple logistic regression analysis OR (IC 95%): 4.4 (1.7–11.2), even accounting for gender adjusted OR [AOR] 4.0, (1.5–10.5); psychiatry family history AOR 3.8 (1.4–10.5); previous suicide attempted AOR 3.7; (1.4–10.5) and Hopelessness AOR 3.2 (1.11–9.4).

**Table 5:** Multiple Logistic regression analysis between depression and childhood emotional abuse among patients.

**Model^a^**	**Childhood emotional abuse**		***p*-value**
	**No**	**Yes–OR (IC 95%)**	
Raw Model	Reference	4.38 (1.71–11.20)	0.002
Model 2	Reference	4.04 (1.54–10.55)	0.004
Model 3	Reference	3.84 (1.40–10.55)	0.009
Model 4	Reference	3.71 (1.31–10.47)	0.013
Model 5	Reference	3.23 (1.11–9.38)	0.031

a *Raw Model: Depression Disorder + Emotional Abuse*.

*Model 2 Raw model + Gender*.

*Model 3 Model 2 + Familiar History of Mental Disorders*.

*Model 4 Model 3 + Suicide Attempt*.

*Model 5 Model 4 + Hopelessness*.

## Discussion

Depression is one of the most common mental disorders, with estimates ranging around 16 % of the population at some point in life. The World Health Organization has recognized that it is one of the psychopathologies that lead to greater commitment and mental suffering, thus being considered a public health problem (36). For this reason, it is a necessary an identification of the etiological factors that could help to minimize the incidence of these conditions and also improve the development of more effective therapeutic approaches to minimize the harmful effects of such a psychopathological condition. The scientific literature has been pointing out that the early stressors in childhood, called early stress, are also closely linked to the onset of psychiatric disorders (4, 37). Among the early stress subtypes, emotional abuse has been studied in several studies, showing that the same appears to be associated with the development of depressive disorders in adulthood (10, 11, 38–40). The study conducted by Pinto et al. (41), with a Portuguese sample also demonstrated, from logistic regression and linear analysis that adversity in childhood and adolescence elucidate around 6% of depressive symptoms and increases the risk of suicide attempts around 1,818 times. In our study evidenced a percentage around 70% of depressive patients with emotional abuse and from the results of the regression analysis was found an odds ratio of 4.38 times of occurrence of depression in patients with emotional abuse in childhood, thus demonstrating the importance of this early stress subtype as a trigger to trigger the depressive disorder. Emotional abuse often becomes unnoticeable by the health and education professionals because of its invisibility and discretion; however, it is imperative to its recognition. Moreover, in our research, we found a predominance of depressive patients were female (72.8%) and from the multiple logistic regression analysis was shown that patients with emotional abuse in childhood and females have an odds ratio of occurrence 4.04 times in developing depression, thus corroborating evidence from the scientific literature. According to studies by Kessler et al. (42), Fleck et al. (43) there is a relationship of two depressed women to a man, from adolescence, prevalence this explained by multifactor, with emphasis neuroendocrine and biological agents. Hormonal changes at different stages of development of women, i.e., premenstrual period, pregnancy and perimenopausal period seem to offer explanations for the association between depression and females (44). Research conducted by Weissman and Klerman (45) pioneered this relationship is particularly important for other researchers to extend understanding of the etiology for the higher prevalence of depression in women compared to men. Furthermore, studies show that men tend to have coping strategies to externalized depression, such as alcohol and drug abuse, while women internalize the symptoms, expressing sadness from social isolation, crying, and emotional withdrawal, more symptoms characteristic of depressive symptomatology (46). Other studies show that adolescent girls have problems related to the sense of well-being, the performance of social roles, as well as physical and sexual abuse in childhood, while men are involved with the abuse of alcohol and drugs, being this behavior a trigger also for attempted suicide (47). Another result of high scientific evidence concerning the association between depression and family history of psychiatric disorder. In our survey, 77% of patients had a family history of psychiatric disorders, and from the regression analysis, patients with a history of emotional abuse, female and mental disorder family history have an odds ratio of 3.84 times more on developing depression. This result corroborates the findings in the literature since the risk of depression increases 2 to 3 times in first-degree relatives of individuals with major depression compared to healthy people (48). Levinson (49) emphasizes that an individual who has a first-degree relative with depression have a higher risk of developing the same disorder, therefore the prevalence of depression in children of depressed parents is 5–12% higher than in the general population. Besides the positive family history is indicative of poor prognosis in psychiatric treatment (24, 50).

In the meta-analysis of Sullivan et al. (51) has seen an odds ratio of 2.84 times in patients with a family history of depression, compared to individuals with no family history. Such genetic influence becomes more evident when analyzing the appearance period of the disease and the number of episodes, as family members of individuals with recurrent depression and early onset present 17.4% rate while the relatives of individuals with single episode late-onset depression and have rates of 3.4% (52). It is worth mentioning that genetic predisposition and interaction with the environment is an individual aspect, therefore will depend on the combination of genetics and environment for the person presenting or not depression (53). Consecrated studies and current also show a potential association between depression and suicide, and impulsive behavior and aggression are important clues in this association (54–57). The study of Isometsa et al. (58) showed that while half of the depressed who committed suicide were receiving psychiatric treatment, few were under proper treatment for depression at the time of suicide, deserving emphasis on male patients, which they receive less frequent diagnosis of depression, thereby receiving less treatment and those who were diagnosed adhere less. Kessler et al. (59) evaluated the ratio between major depression and suicidal ideation, planning, trial and suicidal gestures in the years 1990–1992, and this research repeated after a decade; the findings of this study remained similar, demonstrating the temporal stability of the association between depression and suicide. Our study is in accordance with scientific evidence (24), since more than 70% of the sample had a history of suicide attempt; besides from the statistical analysis it was shown that patients with emotional abuse in childhood, female, with a history of mental illness in the family and attempted suicide have an odds ratio of occurrence of 3.71 times to develop depression. Also, the cohort of 3,017 individuals conducted by Brezo et al. (60) found an odds ratio of 6.8 times between childhood sexual abuse and suicide. Another exciting point identified in this study was the association between depression and hopelessness. According to the theory of Aaron Beck, depression result from thoughts and distorted beliefs where there is a negative view of the future, generating great hopelessness about life itself. Also, the hopelessness is closely associated with suicidal ideation, and the incidence of suicide in depressed people is 15%. Leahy (61) points that stress is a primary source of distorted thoughts in depressed activation in this sense the cognitive restructuring process will work to prevent the presence of depressed mood, apathy, fatigue, and other physiological changes present in depression. In our study, it was clear that patients with emotional abuse in childhood, female, with a history of mental disorders and suicide attempts and hopelessness have the odds ratio of 3.23 times in developing depression. It is important to emphasize that our study presents a methodological limitation regarding the research of early stress with psychiatric disorders in adults, so it would be necessary to evaluate the stress of current life, so further research can help to broaden the understanding of past stress and present, favoring the understanding of depressive illness. Depression is a complex disease, and explained by the interaction of genotype including heredity, the lived-in childhood environment, which can be marked for possible traumas, temperament that gives the individual the ability to deal with the environment and the resilience of some individuals who may explain different response to the same stressful events.

## Conclusions

Our results suggested that the occurrence of depression in adulthood is related to situations of emotional abuse, sexual and physical neglect during childhood. The relationship between depression and childhood emotional abuse in females with psychiatry family history and previous suicide attempted is significant. Thus, these findings contribute to better understanding the mechanism involved in the pathogenesis of depression related to childhood emotional abuse.

## Author Contributions

MJ conceived and designed the study. CM-M, TP, and CB organized the database. MJ, CM-M, ST, and ER performed the statistical analysis and reviewed the literature. CM-M, ST, and CB dealt with psychometric evaluation. MJ, TP, and CM-M wrote the manuscript. MJ critically reviewed and finalized the paper. All authors contributed to manuscript revision, read, and approved the submitted version.

### Conflict of Interest Statement

MJ is Honorary Consultant at South London and Maudsley NHS Foundation Trust. MJ has within the last year received honoraria for speaking from GSK, Lundbeck and Pfizer. The remaining authors declare that the research was conducted in the absence of any commercial or financial relationships that could be construed as a potential conflict of interest.
